# A model of co-creation: strengthening primary health care (PHC) in Ghana through an innovative “Nyansapo” partnership

**DOI:** 10.3389/fmed.2024.1400850

**Published:** 2024-12-09

**Authors:** Princess Ruhama Acheampong, Kulamakan Kulasegaram, Kofi Akohene Mensah, Marie-Therese Ndiaye, Wilberforce Owusu-Ansah, Ellis Owusu-Dabo, Joseph Owusu, Jamie Rodas, Katherine Rouleau, Jennifer Wilson, Olivia Wilson

**Affiliations:** ^1^Kwame Nkrumah University of Science and Technology, Kumasi, Ghana; ^2^Department of Family and Community Medicine, University of Toronto, Toronto, ON, Canada; ^3^Office of Vice President International, University of Toronto, Toronto, ON, Canada; ^4^School of Medicine, University College Dublin, Dublin, Ireland

**Keywords:** Global Health (MeSH [H02.403.371]), Sustainable Development Goals - SDGs, family medicine and primary care, health systems (source: MeSH NLM), higher education institutions (HEIs), co-creation activities, primary health care (MeSH [N04.590.233.727]), global health partnership

## Abstract

The Africa Health Collaborative (AHC) initiative embarked on a transformative ten-year collaboration with Kwame Nkrumah University of Science and Technology (KNUST) and the University of Toronto (U of T) to co-create continuing education programs geared toward augmenting the proficiency of primary care practitioners in Ghana. While upholding core principles within the AHC framework, emphasizing respect, inclusivity, equity, reciprocity, ethics, dynamism, and stewardship, seven teams of U of T and KNUST faculty engaged in collaborative efforts to design, administer, and evaluate five in-person “short courses” in Ghana on Palliative Care, Quality Improvement for Health Professionals, Prehospital Emergency Care, Community Emergency Care, and Emergency Preparedness and Response to Epidemic-Prone Diseases to approximately 100 Ghanaian primary care professionals. This paper describes a model of co-creation, highlights lessons learned from a robust evaluation process, and proposes that this co-creation model can strengthen primary health care in Ghana and ultimately transform health systems in Africa.

## Introduction

Since 2022, the U of T, KNUST, and seven other African universities have embarked on a ten-year collaborative effort to enhance primary health care (PHC) workforce education, entrepreneurship, and innovation across Africa. The bold initiative is focused on health transformation for Africa by Africans (1).

KNUST is one of the nine AHC partners that aims to contribute to three pillars of the health strategy: Health Employment, Health Entrepreneurship, and Health Ecosystems. In Ghana, the health sector’s success is critical in achieving the Sustainable Development Goals (SDGs), especially SDG3, which aims to “ensure healthy lives and promote wellbeing for all ages.” Despite the government’s support for the health sector, Ghana suffers from a mismatch between the demand for health and the supply of critical health workforce toward attaining universal health care coverage. The paramount objective of KNUST Health Employment Pillar (HEMP) is to co-create and co-facilitate five continuing education programs to build the competency of primary care workers and contribute to health systems that employ and retain the primary care workforce (2).

In October 2022, the U of T’s Department of Family and Community Medicine (DFCM) was invited to collaborate with KNUST’s School of Public Health (SPH) to meet the objective of the HEMP pillar. The target audience is primary care providers (PCPs) such as community health nurses, nurses, clinical officers, and general practitioners with a focus on women. In response to the identified needs in Ghana, KNUST and U of T faculty co-created five “short courses” on the topics of Palliative Care, Quality Improvement for Health Professionals, Prehospital Emergency Care, Community Emergency Care, Emergency Preparedness and Response to Epidemic-Prone Diseases. Between September and December 2023, seven U of T faculty teams traveled to Ghana to co-facilitate these short courses alongside KNUST faculty to over 100 Ghanaian PCPs. Over seventy-five faculty and senior leaders from both institutions were involved in this innovative initiative.

The term co-creation often focuses on “how” to co-create, especially in health and community settings (3); however, ill-defined terms are used interchangeably to reflect co-creation, and a lack of consensus remains on the meaning and use of the term. A literature review has shown that co-creation is conceptualized and operationalized in many ways, even within the same field. In health, the current trend is to depict co-creation as a participatory research model (4, 5). Others define co-creation as the fusion of community-based participatory research and integrated knowledge translation (6), while some researchers base their understanding on a model devised by Sanders and Stappers (7, 8). In the latter example, co-design is described as a collection of activities ranging from ideation to planning and evaluation.

Despite the lack of consensus, two specific definitions of co-creation have been proposed to resolve some of this conceptual ambiguity: (A) “a process whereby researchers and stakeholders jointly contribute to the ideation, planning, implementation, and evaluation of new services and systems as a possible means to optimize the impact of research findings” (9); and (B) “the collaborative generation of knowledge by academics working alongside stakeholders from other sectors” (10). Although both definitions share the concept of equitable collaboration between stakeholders, neither definition captures the concept of co-creation as simultaneously focusing on program or policy delivery and generating new knowledge. The lack of a universally accepted definition creates unnecessary ambiguity (11–14), and researchers cannot effectively search electronic databases and retrieve relevant studies, inhibiting the development of a coherent, critical mass of adequately homogenous co-creation research.

In global health, co-creation between stakeholders from lower- and middle-income countries (LMICs) and high-income countries (HICs) may be a suitable implementation method for equitable research, education, and healthcare interventions (15–18). A literature search revealed studies where co-creation was used to describe collaborations between countries; however, the meaning of co-creation varied. Many studies referred to co-creation as a process occurring between stakeholders and end-users within an institution, region, or country (19–26). Some studies described co-creation as taking resources previously developed by institutions in one country and adapting them to meet the needs of a separate country without close collaboration between stakeholders (16, 18, 27). A co-creation initiative between Harvard Medical School and five medical schools in Vietnam to create, implement, and evaluate new curricula for Vietnamese medical schools was more in line with the first definition of co-creation described above (17). Despite various approaches to co-creation to solve global challenges, it can introduce culturally appropriate and relevant initiatives in LMICs through partnerships and support with HICs (15, 16, 18, 20, 25). Furthermore, global co-creation is aligned with SDG17, promoting partnerships between LMICs and HICs for sustainable and equitable global development. These partnerships hold the potential to both catalyze and facilitate the achievement of the other sixteen goals, including SDG3, which focuses directly on promoting, improving, and sustaining health globally (28).

**Figure 1:**
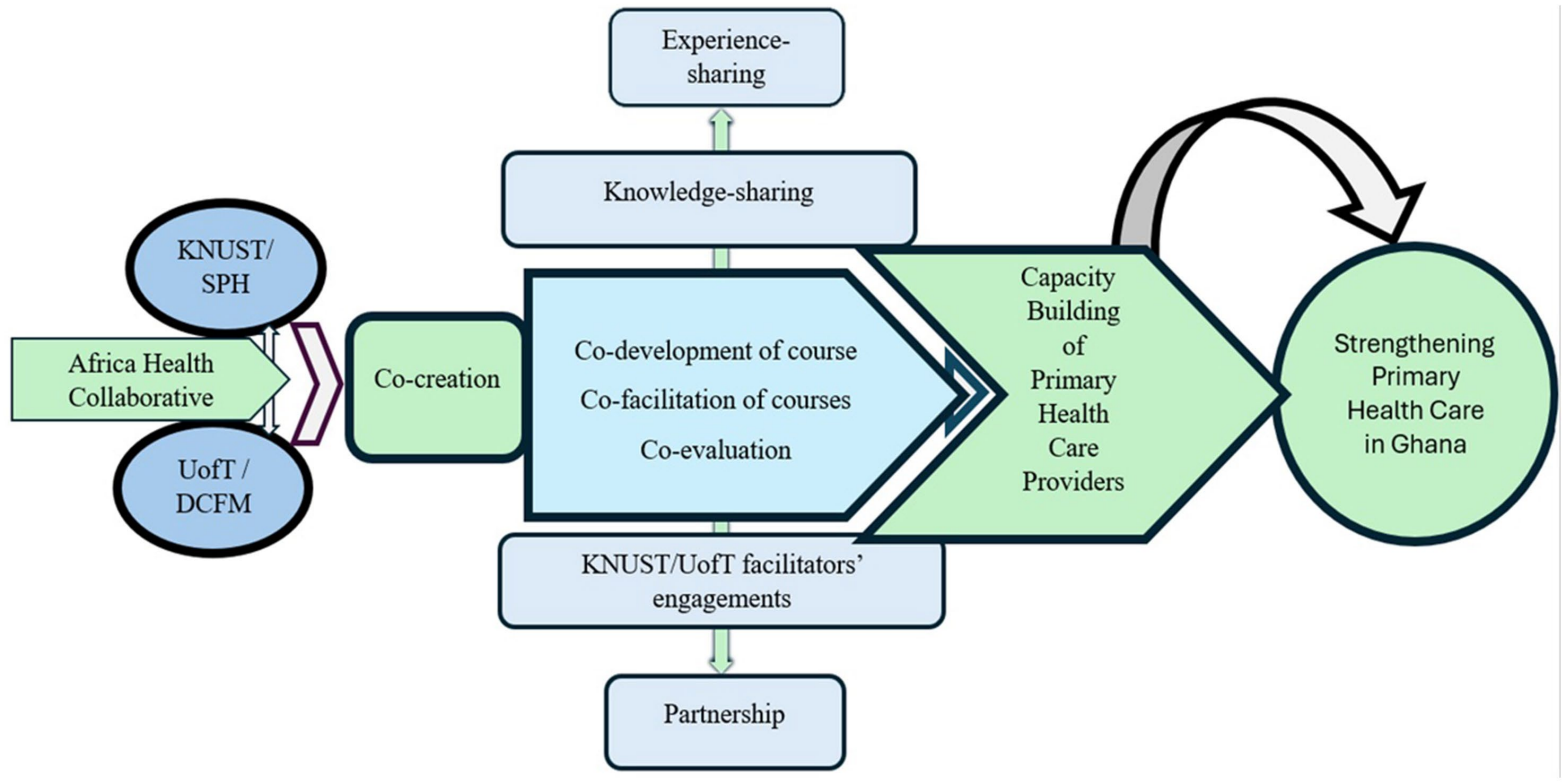
Co-creation model for strengthening Primary Health Care in Ghana between Kwame Nkrumah University of Science and Technology (KNUST) and University of Toronto (U of T)’s Department of Family and Community Medicine (DFCM).

With scholars calling for a consensus on the meaning and approach of co-creation (29), this paper proposes a model of co-creation between a Canadian and an African higher education institution under the Africa Health Collaborative (AHC) (See Figure 1). The authors have chosen not to use the terms North–South or HIC-LMIC to describe this partnership due to the positionality these terms evoke. Instead, we turn to our African colleagues to decolonize our partnership language. “Nyansapo” or the “Wisdom Knot” is a revered Adinkra symbol representing wisdom, ingenuity, intelligence, and patience. This symbol conveys the idea that wise people have the capacity to choose the best means to attain a goal. Being wise implies broad knowledge, experience, as well as the ability to apply such faculties to practical ends (30). A “Nyansapo” Partnership exists at the powerful intersection of local wisdom and global collaboration and holds the potential to benefit those committed to equitable partnerships in global research, education, and health interventions. This model can be replicated and adapted by standardizing co-creation language to better inform others of the international processes that strengthen PHC systems.

## Methods

This innovative co-creation process was guided by the AHC’s shared human rights principles: academic freedom, Africa-centricricity, employability, networks, impact, scale, sustainability, and indigeneity. The faculty from U of T and KNUST strove to live by the AHC’s shared values of respect, inclusivity, equity, reciprocity, ethics, dynamism, and stewardship.

### Stakeholder needs assessment

To document the gaps in Ghana’s healthcare delivery, experts from the Ghana Health Service, higher education institutions, Komfo Anokye Teaching Hospital (KATH), and the Christian Health Association of Ghana (CHAG) were invited to initiate the co-creation process. Through comprehensive and detailed engagement with policy-level stakeholders and implementers nationwide, capacity-building for primary care providers emerged as the top priority for advancing universal health care. KNUST received strong support from the government and health leaders to identify the themes and deliver the short courses.

### Selection of short-course teams

Once KNUST and U of T established their senior leadership teams, short-course leads with subject matter expertise and resources were identified. They swiftly assembled course development teams and defined roles, responsibilities, and timelines. A virtual orientation session was held for all faculty.

### Collaboration and communication channels

Regular and consistent communication was established between KNUST and U of T leadership. Shared document platforms, Zoom meetings, email, WhatsApp messaging, learning circles, and bi-weekly project updates were used to collaborate, keep the collective team organized, and deal swiftly with challenges that arose. These processes preserved equity in sharing resources and were instrumental forums for co-learning.

### Co-creation of short courses

KNUST held short-course retreats to create objectives, identify target audiences, and create an outline for each short course, after which KNUST and U of T short-course teams began meeting online to co-create the course content. Teaching faculty were identified and slides were co-created for course delivery. This process was an essential learning experience for U of T and KNUST faculty members, revealing how the five themes were dealt with in Canada and Ghana, and contextualizing the content of modules based on local needs. A timetable for co-facilitation was established to facilitate the order of the course delivery. All curricula were sent to Ghana’s professional bodies for accreditation: Ghana Medical Council, Nursing and Midwifery Council, Pharmacy Council, and the Allied Health Professional Council. These professional bodies awarded Continuous Professional Development (CPD) points to successful participants.

### Co-creation of monitoring and evaluation plan

DFCM’s Office of Education Scholarship (OES) helped co-create an evaluation plan including end-of-course surveys, interviews, and focus groups was designed to assess and guide improvements for the five short courses. Workshops were held with the KNUST/U of T leadership team after which an evaluation strategy was co-created to engage teachers and learners to understand the learning experience in these new courses (31, 32) (see Supplementary material).

### Course promotion

Flyers containing course eligibility, application guidelines, and contact numbers were designed by KNUST for each course and circulated via the media, university website, email, and social media handles (WhatsApp et al.) in Ghana. Announcements were made regarding the short courses during stakeholder meetings, health conferences, and engagements. Primary care providers in disadvantaged communities in partner institutions of the AHC were also invited through the AHC communications team.

### Participant selection

Prospective applicants applied through the KNUST website to receive a scholarship to support their participation. An independent team of short-course facilitators and staff of the AHC and KNUST shortlisted participants. A score sheet was designed based on gender, cadre, practice location, motivation, and years of experience. The first 20 participants with the highest percentage in order of ranking were selected and sent congratulatory emails and phone calls. Unsuccessful applicants were informed of the outcome by email.

### Preparation for launch

Preparations for the U of T faculty’s departure to Ghana included orientation, the creation of a travel manual, booking of flights and hotels, and application for a scholarly travel visa. In order to track progress and problem-solve promptly across time zones, traveling teams remained constantly connected with KNUST and U of T leadership via a WhatsApp group. KNUST’s outstanding logistical and security support and exceptionally warm hospitality were major enablers for the success of each short course.

### Course delivery

Each course began with an opening ceremony that sought to formally welcome facilitators from Ghana and Canada, faculty of KNUST, staff of the AHC, course participants, and organizers. AHC and KNUST leadership made presentations on their vision and strategy. U of T course leads also remarked about their enthusiasm for contributing to health care delivery in Ghana. These presentations and remarks were necessary to enlighten both teams on the co-creation strategy of the AHC and to consider other relevant sectors in health care delivery for co-creating in the future. KNUST, led by the Pillar Lead and International Liaison Officer, formed a committee of four to coordinate activities during the delivery of each course. These included site logistics, internet connectivity, stationery, and emergency requests.

### Monitoring and evaluation

The evaluation team at DFCM’s OES trained a KNUST monitoring and evaluation team via three Zoom meetings. Both teams discussed the data collection strategies, transcriptions, analysis, and KNUST strategies to monitor and evaluate these courses in the future. The KNUST team administered end-of-course surveys and conducted the interviews and focus groups after which all audio files were shared with U of T’s evaluation team for transcription and analysis. This quantitative and qualitative data allowed for triangulation of learner experience and identified particularly effective learning moments from the teacher’s perspective. Supplementing this data are structured observations from trained observers (see Supplementary material).

### Closing short course delivery

On the last day of the delivery, all short courses were formally closed with a brief ceremony. The last day was named “Ghana Day.” It was a day to wear custom-designed Ghanaian clothing and eat Ghanaian dishes. It was also a time for sharing individual and team successes, and awarding certificates to deserving participants who had completed the short courses. All closing ceremonies featured a taste of Ghanaian culture and dance. This was necessary to celebrate the success of each short course and create a unique bond between faculty and learners on both teams.

### Post-short course debrief

After course completion debriefs, faculty were treated to sightseeing in Ghana hosted by KNUST faculty. After a tour of the spectacular KNUST campus, teams traveled to Cape Coast to visit a KNUST training facility and Kakum National Park. The final excursion took teams to Elmina Castle, which has been preserved as a World Heritage Monument under UNESCO. As academics who study and teach structural determinants of health, U of T faculty were left with much to reflect upon and were challenged by the tour guide’s final two words as they stepped away from the Door of No Return. *“Never again,” he stated. “Never again,” he repeated.*

## Results

All partners co-created an extensive qualitative and quantitative evaluation report (see Supplementary material). Highlights include:

### Co-creation and co-delivery experience

The evaluation results indicated that faculty from both DFCM and KNUST were very positive about the co-creation experience and working with diverse colleagues. The opportunity for sharing knowledge, experience, and national/local context was thought to be an important part of successful education and to create a better learning experience. Faculty valued the diversity of their teams and the flexibility and adaptive approach to co-creation. They also noted the added value of co-creation in producing new understanding and knowledge of different knowledge and expertise areas. Lastly, faculty noted that a shared sense of values and mission enhanced this work (see Supplementary material).

## Learner experience

Overall, from the learner perspective, the pedagogical design and delivery of the short courses met the intended goals of each course and the overall program. Across all courses, participants reported feeling highly satisfied, grateful, and knowledgeable in the goals of the courses as expected. Participants demonstrated a strong likelihood of recommending the short courses to their peers, expressed interest in further training, and advocated for broader dissemination of course content. They also expressed the need to de-localize the training to hard-to-reach areas. The courses were praised for their effective teaching methods and ability to meet learning objectives. Moreover, learners recognized that the ability to influence change and impact the healthcare system would depend on the coordination of colleagues, leaders, and all stakeholders in the healthcare system (see Supplementary material).

## Discussion

From the comprehensive evaluation of the collaborative educational program, several key lessons have been learned:

1. Importance of co-creation: the co-creation process, though challenging, proved essential in harnessing diverse insights and expertise, responding to local needs, and ultimately enriching the educational content. The engagement of multiple stakeholders led to a more tailored and contextually relevant learning experience.
2. Flexibility and adaptability in course design and delivery: flexibility in adapting course content for health professionals working in low-resource settings and to the varying backgrounds and skills of participants was crucial. This adaptability needs to be maintained and enhanced in future programs to accommodate the diverse needs of participants and to handle unforeseen challenges more effectively.
3. Effective engagement and communication: regular and structured communication among course designers helped in maintaining project momentum and alignment. Future programs may require more time for co-creation, engagement in team meetings, and clarity on roles, responsibilities, and timelines.
4. Building on success and addressing practical needs: the high levels of participant satisfaction and engagement indicate that the courses met their primary educational goals. A recurring theme across the courses was the need for more practical, hands-on learning experiences. This feedback underscores the importance of integrating more simulation- based/field work and interactive components to continue to bridge the gap between theoretical knowledge and practical application.
5. Longitudinal impact and stakeholder coordination: the longitudinal nature of some modules allowed for the development of ideas and better preparation for practical application. Faculty provided mentorship and support to participants between modules and after course completion as they implemented their projects. Future programs should consider the benefits of extending this approach to foster deeper learning and more sustained impact.
6. Delocalization and social inclusion: some course participants traveled long distances to attend courses in Kumasi. Plans to delocalize the short courses and delivery to create more equitable access to rural areas are underway. While 80% of course participants were young women, this key target audience faces many barriers to attending courses including pregnancy-related and childcare needs. Efforts to identify and address these barriers are being prioritized.

## Conclusion

KNUST and U of T have modeled co-creation through a “Nyansapo” partnership to project the complementary strengths of global partners to achieve the bold collective goal of strengthening Ghana’s PHC system by strengthening the capacity of primary care workers through educational courses. Drawing from lessons learned, the KNUST/U of T strategy for co-creation holds excellent potential to leverage sustainable change, catalyze SDGs, and create platforms that lead to health transformation for Africa. This KNUST/U of T co-creation approach could be replicated and adapted for global health partnerships between organizations, teams, and higher education institutions.

**Figure 2:**
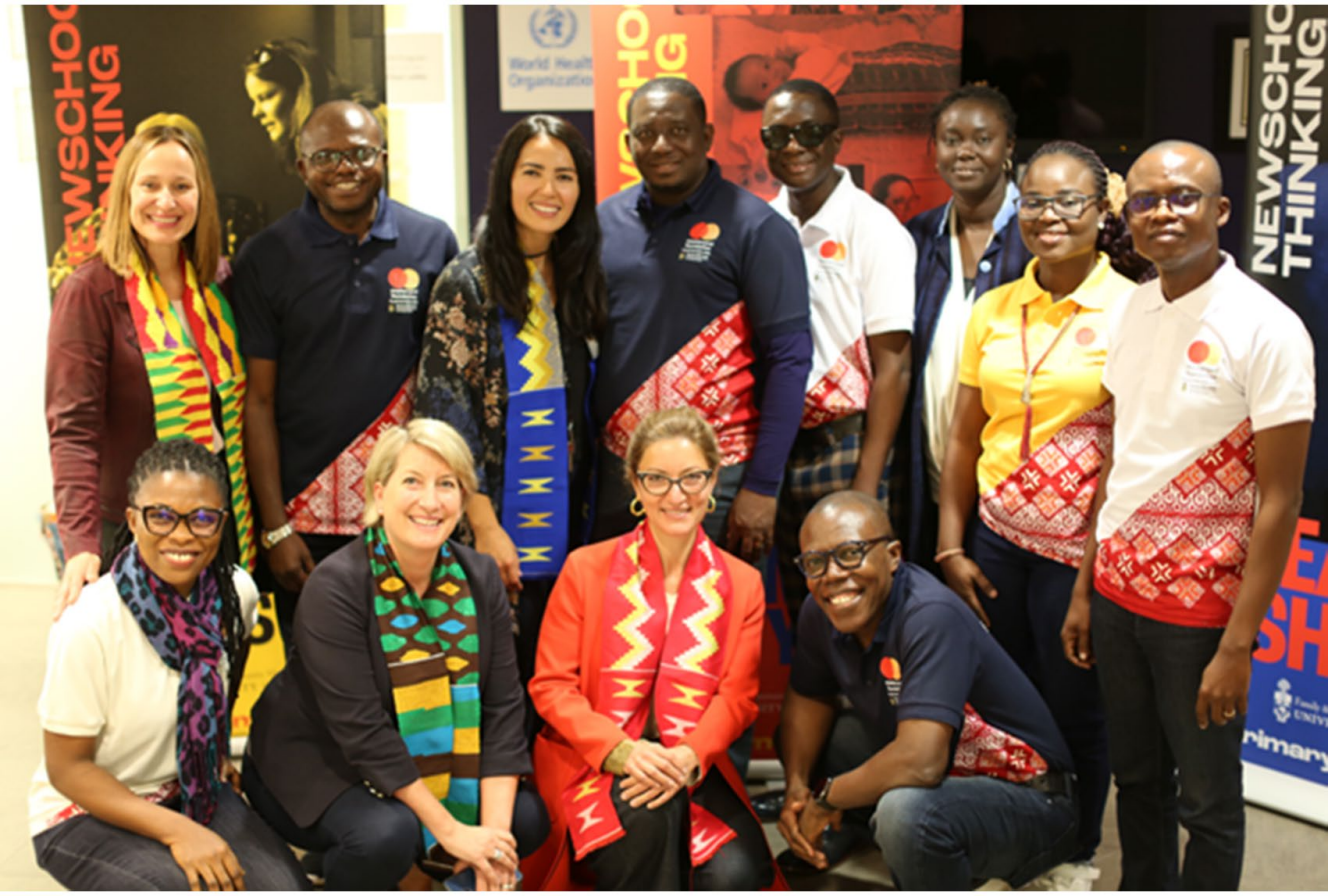
Top Row (Left to Right): Dr. Jennifer Wilson (Department of Family and Community Medicine, Faculty Partnership Lead, University of Toronto), Prof. Wilberforce Owusu-Ansah (Health Entrepreneurship Pillar Lead, Kwame Nkrumah University of Science and Technology), Jamie Rodas (Department of Family and Community Medicine, Global Health Coordinator, University of Toronto), Mr. Emmanuel Ebo Ocran (Finance Manager, Kwame Nkrumah University of Science and Technology), Dr. Kofi Akohene Mensah (Health Employment Pillar Lead, Kwame Nkrumah University of Science and Technology), Marie Therese Ndiaye (Office of the Vice-President, International, University of Toronto), Dr. Princess Ruhama Acheampong (Liaison Officer, Kwame Nkrumah University of Science and Technology), Dr. Joseph Owusu (Health Ecosystem Pillar Lead, Kwame Nkrumah University of Science and Technology), Bottom Row: Mrs. Eva Boakye-Yiadom (Project Manager, Kwame Nkrumah University of Science and Technology), Dr. Katherine Rouleau (Global Lead Primary Health Care, Office of Health Systems Partnerships, University of Toronto), Dr. Danielle Martin (Chair, Department of Family and Community Medicine, University of Toronto), Prof. Ellis Owusu-Dabo (Principal Investigator & Pro-Vice Chancellor, Kwame Nkrumah University of Science and Technology).

One of the surprises of co-creation is the personal, professional, and institutional transformation taking place in both institutions as we “untie the wisdom knot” together. As partnership values and principles are upheld, and as this “Nyansapo” partnership deepens and matures, these transformative changes will continue to be documented and disseminated (Figure 2).

## Data Availability

The original contributions presented in the study are included in the article/Supplementary material, further inquiries can be directed to the corresponding authors.
