# Supplementary material for: A model of co-creation: strengthening primary health care (PHC) in Ghana through an innovative “Nyansapo” partnership
Source: Front Med (Lausanne). 2024 Dec 9;11:1400850. doi: 10.3389/fmed.2024.1400850 (PMC11663670; doi:10.3389/fmed.2024.1400850)
Supplement: Supplementary file 1 [file Data_Sheet_1.PDF]

Evaluation Report

KNUST-DFCM Short Courses 2023

Date of Submission: May 31, 2024 (Updated August 22, 2024)

Table of Contents

LIST OF ABBREVIATIONS....3

EXECUTIVE SUMMARY 3

INTRODUCTION....4

EVALUATION APPROACH 5

EVALUATION METHODS....6

RESULTS 9

INTEGRATION OF RESULTS 28

LESSONS LEARNED 29

REFERENCES....30

APPENDICES 31

List of Abbreviations

AHEHC: Africa Higher Education Health Collaborative
CEC: Community Emergency Care
DFCM: Department of Family and Community Medicine
EMPRESS: Emergency Preparedness and Response to Epidemic-Prone Diseases
KNUST: Kwame Nkrumah University of Science and Technology
M & E: Monitoring and Evaluation
OES: Office of Education Scholarship, University of Toronto
OVPI: Office of the Vice-President International, University of Toronto
PC: Palliative Care
PEC: Prehospital Emergency Care
QI: Healthcare Quality Improvement for Healthcare Professionals (QI)
ToC: Theory of Change
U of T: University of Toronto

Executive Summary

The Africa Higher Education Health Collaborative (AHEHC) is a network of nine African institutions, including the University of Toronto, aimed at enhancing African health sectors through cross-border and cross-sector partnerships. The initiative focuses on three pillars—health employment, health entrepreneurship, and health ecosystems—with the Department of Family and Community Medicine (DFCM) at the University of Toronto and the School of Public Health at the Kwame Nkrumah University of Science and Technology (KNUST) collaborating under the health employment pillar to develop continuing education programs that build the capacity and skills of primary health care workers in Ghana.

Supported by the Office of Education Scholarship (OES), a comprehensive evaluation plan, including end-of-course surveys, interviews, and focus groups, was implemented to assess and guide improvements of the short course programs. The evaluation results indicated high satisfaction among learners, with the pedagogical design and delivery meeting the intended goals of the courses. Key lessons learned include the importance of co-creation for tailored and contextually relevant learning content, maintaining flexibility in course design, effective communication for project alignment, and incorporating more practical, hands-on learning experiences.

Introduction

As part of the Africa Higher Education Health Collaborative (AHEHC) initiative, the Department of Family and Community Medicine (DFCM) at the University of Toronto (U of T) in Canada was invited into a ten-year collaboration with Kwame Nkrumah University of Science and Technology (KNUST) in Ghana. KNUST is one of the ten Health Collaborative partners that aims to contribute to three pillars of the health strategy: Health Employment, Health Entrepreneurship, and Health Ecosystems.

These programs will contribute to attaining the Sustainable Development Goals (SDGs) 3, 4, 5, 8, 9, and 17 in Ghana and across Africa. In Ghana, the health sector's success is critical in achieving the SDGs, especially SDG3, which aims to "ensure healthy lives and promote wellbeing for all ages." Despite the government's support for expanding and improving the health sector, Ghana suffers from a mismatch between the demand for health care and services and the supply of critical health workforce towards attaining universal health care coverage.

It was a high honor for DFCM to accept this partnership invitation to collaborate with global partners in advancing high-quality primary and emergency care around the world. In line with DFCM's strategic plan, this collaboration provides a rich opportunity for our faculty and leadership to learn from our Ghanaian colleagues and live out our values by engaging in an equity-focused, practical, needs-responsive North-South partnership. Beyond the inherent value of collaboration, the partnership further presents a rich potential to catalyze and inform sustainable change and health system transformation in Ghana and in Canada.

The paramount objective of the KNUST-DFCM collaboration under the Health Employment Pillar (HEMP) is to co-create and co-facilitate five continuing education programs to build the competency of primary care workers to meet the growing demand in Ghana and contribute to health systems that employ and retain the primary care workforce. The partnership is informed by ethical principles including solidarity, humility, cultural sensitivity, respect, equity, reciprocity, and shared accountability.

Through extensive stakeholder engagement across Ghana, KNUST undertook a needs analysis to identify areas of health services that presented opportunities for strengthening. In response, seven teams of KNUST and DFCM faculty co-created and co-facilitated five inaugural in-person short courses in Palliative Care (PC), Healthcare Quality Improvement for Healthcare Professionals (QI), Prehospital Emergency Care (PEC), Community Emergency Care (CEC), and Emergency Preparedness and Response to Epidemic-Prone Diseases (EMPRESS). The development and delivery of these five short courses is also referred to in this document as “the program”. The following Co-creation model guided the development, delivery, and evaluation of the education programs:

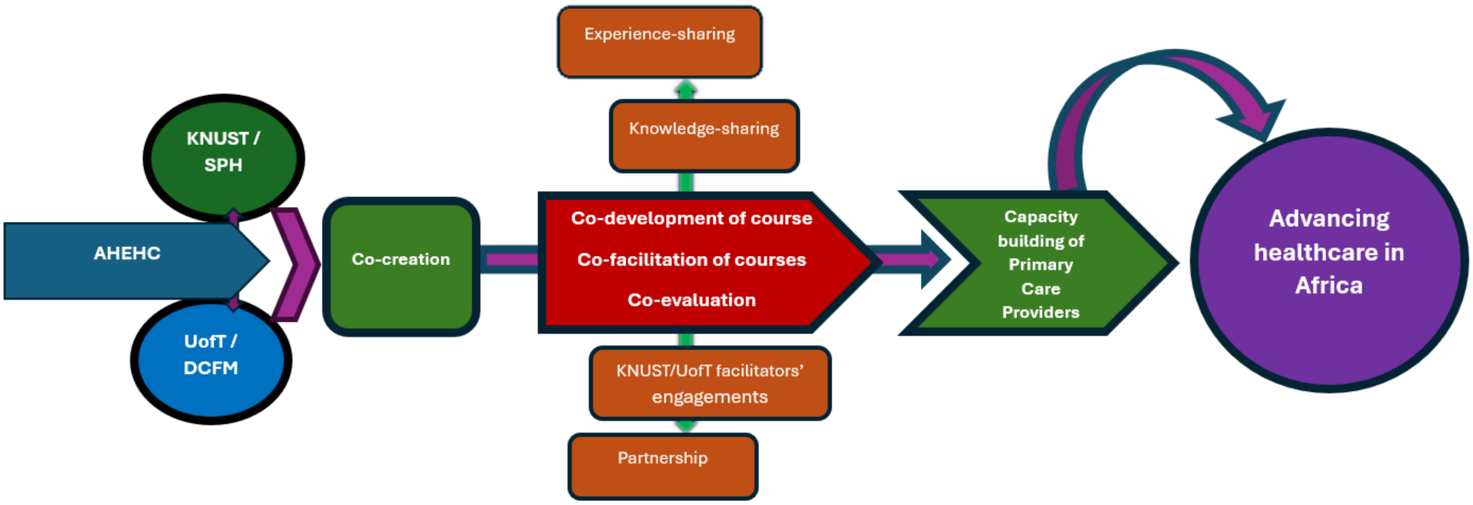

KNUST/U of T Co-creation model for strengthening PHC in Ghana[1]

Supported by the Office of Education Scholarship (OES), a robust monitoring, evaluation, learning, and adaptation plan was co-created, and included end-of-course evaluation surveys, teacher interviews, and learner focus groups. The evaluation of this project is crucial to ascertain its effectiveness, identify areas for improvement, and guide future educational endeavors. This report outlines the methodologies employed in the evaluation process, highlighting the collaborative efforts of the organizational leadership team at KNUST and DFCM with a focus on implementing a robust educational assessment framework.

This evaluation's primary goal was to document the pedagogical experiences of creators, facilitators, and learners using a framework of intended and emergent processes. Secondarily, the evaluation captured preliminary and self-reported evidence of learning outcomes and application of knowledge.

Evaluation Approach

The approach for the evaluation of this program was inspired by Haji F, Morin MP, and Parker K (2013)[2] and colleagues who outline the importance of process-based evaluation that captures both intended and emergent processes. In this view, while outcome evaluation remains important, the processes by which outcomes are derived are equally important as they contribute to understanding how outcomes can be optimized in the future through process changes. Moreover,

the critical elements of emergent and unintended processes and outcomes must also be submitted for evaluation. This requires that evaluation methods and syntheses move beyond a typical 'quick win' outcome evaluation and use qualitative methods to understand the experiences and perceptions of learners and faculty.

Therefore, this evaluation was focused on the pedagogy of the short course experience and the perceived learnings from the perspectives of the participants and faculty. External outcome measures of learning and impact were not part of the evaluation. Furthermore, specific course level impact was not a target of the evaluation but rather contributed to an evaluation of the whole program. However, some course specific learnings are documented in this report as emergent processes and outcomes.

Evaluation Methods

The methodology used for the evaluation of the KNUST-DFCM educational collaboration program included three principal stages: evaluation framework development, evaluation of the course creation process, and evaluation of the course delivery and learning experience. Each stage was designed to provide a comprehensive understanding of both the process and impact of the educational program, utilizing a mix of qualitative and quantitative data collection methodology.

1. Evaluation Framework Development

Objective: To establish a Monitoring and Evaluation (M&E) framework to guide the overall evaluation process.

Methodology: The Office of Education Scholarship (OES) led two co-creation workshops involving key stakeholders from, KNUST, DFCM and the University of Toronto' Office of the Vice-President International (OVPI), which functions as the Secretariat for the Health Collaborative. The purpose of this workshop was to collaboratively develop a comprehensive M & E framework that would address the specific needs and goals of the program.

Outcome: The workshop resulted in a tailored M & E framework that outlines the key components of the evaluation, including objectives, methodologies, and expected outcomes. This framework served as the foundational document guiding all subsequent evaluation activities. In addition to the development of the M & E framework itself, this process further highlighted a number of key commonly shared views including the importance of focusing on co-creation, the desire for productive scholarship resulting from this process, and the wish to produce useable findings to inform future collaborations.

2. Evaluation of the Course Co-creation Process

Objective: To outline the Theory of Change (ToC) for the educational program, including its goals, processes, activities, outcomes, and outputs. Additionally, to extract lessons that could inform other projects and contribute to capacity building in health education.

Methodology: This stage involved detailed interviews with organizational leadership from DFCM, KNUST, and OVPI and faculty leads from DFCM and KNUST. We developed an understanding of the process of co-creation for the evaluation, focusing on aligning the program's broader objectives with the specific goals of the sub-courses or modules.

Outcome: The evaluation provided a detailed description of the course creation process and outlined the critical ingredients of successful co-creation. The evaluation revealed the value of co-creation from the perspective of course leads and identified some logistical barriers and key enablers such as shared values and team diversity. Additional outcomes also included knowledge-sharing activities about co-creation processes through poster and oral presentations at academic conferences and the submission of a manuscript to a peer-reviewed journal.

3. Evaluation of Education Implementation and Experience

Objective: To implement a multipronged evaluation of the courses and modules of the education program. The evaluation sought to understand the experiences of faculty and course participants, identifying effective and ineffective learning activities.

Methodology: An evaluation survey was administered both before and after the education sessions. Participant observation techniques were used during one set of learning sessions, and interviews or focus groups were conducted with participants and faculty after course completion. Participants were also recruited for Module 2 focus group follow-up in the Palliative and QI courses.

Outcome: The data collected provided insights into the effectiveness of the learning activities and mechanisms, offering suggestions for improvements in future iterations.

Each of these stages was critical in providing a thorough understanding of the educational program, helping to ensure that the interventions were not only effective but also adaptable for future educational initiatives.

Each phase of the evaluation was designed to ensure a comprehensive analysis of the educational interventions, with a focus on ongoing improvement and the program's sustainable delivery. The collaborative method, involving diverse stakeholders, not only enhanced the evaluation process but also amplified the overall effectiveness of the educational initiative on primary healthcare in Ghana.

Recruitment

All learners (N=100) were invited to complete an evaluation survey at the end of each course. Evaluation surveys were anonymous and asked basic demographic and experience questions. Learners in courses with two modules (QI n=12 & PC n=10) were invited to participate in focus groups at the beginning of the second module. Learner recruitment was carried out by KNUST faculty who functioned as data collection leads. WhatsApp groups which were used to facilitate recruitment and were maintained by course participants for ongoing collaboration and mentorship. Faculty members who participated in co-design and facilitation were recruited in person and via email for interviews by the data collection team. We aimed for a sample of both KNUST and DFCM faculty to adequately capture the experiences of both teams with the aim of at least 2 teachers per course included in the sample (N=38).

Data Collection Tools

The evaluation survey tool had 18 questions (four open-ended) and a mix of response types. Likert scales were used to rate the overall experience and self-assessed learning outcomes while open-ended questions allowed respondents to summarize learnings and provide more general feedback. The evaluation tool was edited and modified by each course's leads though the overall format and questions were kept generally consistent to allow comparability and general summary of the short course program. Surveys were delivered using Qualtrics®, a software platform designed for managing and analyzing data. Course leads shared the survey link with all course participants who completed the survey on their personal cell phones.

Interview and focus group guides were refined with input from KNUST and DFCM stakeholders and input from the KNUST data collection team. Each guide was semi-structured to allow for variation in responses and emergent themes. Responses were recorded, anonymized and transcribed by the data collection teams prior to analysis.

Analysis

Analysis of surveys and focus-group interviews was done using descriptive statistics and summaries of open-ended responses using thematic analysis. We did not engage in any inferential statistics because of low sample size across courses (<30 individuals) and lack of variance in responses. Qualitative data was analyzed using a qualitative analysis approach which allows for both inductive and deductive summation of data into themes[3]. This data was then further analyzed to extract lessons learned and insights gained by participants.

Results

Participant recruitment was particularly effective as we received nearly 99% response rates to evaluation surveys from all participants. Participant recruitment for qualitative data collection is reported in Table 1 below.

Table 1: Qualitative Data Collection Summary Table

Course	Interviews with Faculty	Focus Groups
Community Emergency Care (CEC)	8	-
Prehospital Emergency Care (PEC)	9	-
Emergency Preparedness & Response to Epidemic-Prone Diseases (EMPRESS)	8	-
Palliative Care (PC)	7	2 (12 participants)
Healthcare Quality Improvement for Healthcare Professionals (QI)	6	2 (10 participants)
Total	38	4 (22 participants)

The results below are organized according to the three components of the evaluation. We first present a high-level overview of the co-creation process. Secondly, we provide an overview of learner experience with a specific focus on QI and PC using qualitative data from the focus groups. We then triangulate evidence and learnings from facilitation.

Co-creation

Responses to DFCM and KNUST faculty interviews about co-creation provide insight into the process of creation of the courses and the latent program theory and intentions of the short courses. While each short course involved its own unique experiences, several common themes emerged from interviews. These are broadly grouped into two major areas: 1) Experience of co-creation and 2) Intended Design.

Co-Creation Experience

Faculty from both DFCM and KNUST were very positive about the co-creation experience and working with diverse colleagues. The opportunity for sharing knowledge and experience was thought to be an important part of successful education and to create a better learning experience. Participants valued the diversity of their teams and the flexibility and adaptive approach to co-

creation. Faculty also noted the added value of co-creation in producing new understanding and knowledge of different knowledge and expertise areas. Lastly, participants noted that shared sense of values and mission enhanced this work. These themes with representative quotes are further explained below.

1. Team dynamics and team diversity: Clear roles and responsibilities within co-creation projects are important to avoid confusion and overlap. Understanding each team member's work style and adapting to team dynamics are essential for effective collaboration. In this instance, co-creation projects involved teams of healthcare professionals from, Ghana, and Canada, brought together to share ideas and develop education content, ultimately to improve patient care. This diversity led to a rich exchange of knowledge and experiences while negotiating team roles and different expertise areas. This also created opportunities for shared learning as skills and knowledge were shared among diverse team members. There was also genuine appreciation between team members for diverse expertise areas and assumption of leadership.

   "And those initial stages were quite tricky. You didn't know how to approach each other, I would say, but over time, once the understanding builds things just to go from there, I worked with, call it with XXX…And I ended up putting those materials together with XXX and the next meeting and he was rather praising me for having done all these things separately, you contributed this much to it…. So, for me, any chance which brings two or more people with diverse experiences together, is an opportunity to latch on. Because there is always thought that give and take moments where both parties end up picking so much out of..." ..." Ghanaian Faculty Member

2. Flexibility and adaptability: Course design required a balance of flexibility in approach and clear deadlines, allowing teams to adapt to various circumstances like the availability of teachers, professional and cultural knowledge, and personal preferences. This flexibility helps to manage the diverse nature of collaborative efforts. Flexibility, letting go of control, and open-mindedness are repeatedly emphasized as crucial for successful co-creation. These attributes allow for better integration of diverse inputs and adaptations to changing circumstances or feedback during the course development.

   "I mean, I think the biggest thing that I would say would be flexibility, and like cultural humility, because, again, there were things that we would do a certain way in our context, that it was clear would not work as well here. And so, we need to (...) be flexible, both in terms of adding things and in terms of taking away things compared to what we would usually do (i.e. in home context)." Canadian Faculty Member

3. New insights and knowledge transfer: In global health, this co-creation project brings unique insights from diverse cadres of health professionals, contributing to a deeper understanding

of learner needs as well as possible methods of teaching and service delivery. Co-creation significantly impacts knowledge transfer, helping to tailor learning experiences to specific needs and contexts. This knowledge transfer was perceived as being unique in that it created 'new' ways of teaching and delivering healthcare that might not otherwise exist. This process of sharing, adapting, and then transferring was identified by both Canadian and Ghanian faculty as a unique advantage of co-creation.

"I believe co-creation has taught me how to function in a diverse world, and how to assess and tap into the knowledge as well as the experience of others, bring on board my own experience in my own knowledge, and then mix them up together and come up with something beautiful as I mean, this goes I think it was something that I'll never forget." Canadian Faculty

"So, a lot of important transfer of knowledge, and interactions. And then they also learn what we are doing here and try to make suggestions that can also improve our system. And then tell us what they also do differently there. So that we can also look at it moving forward and see whether we can also incorporate that into our system." Ghanaian Faculty

"When you co-create, it helps you to have more insights. The fact is, usually we are used to what we see around us. So, if you have someone coming in from the outside, the person might even identify certain things that you have noticed yourself, or the person is coming with a fresh perspective, a fresh look contents, or even a tried and tested different approach to managing things. I believe that's what's unique about co-creation" Ghanaian Faculty

4. Mutual respect and shared values: The foundation of successful co-creation in health care education. Conversely education is built on shared responsibility, mutual respect, and a common goal of improving patient care. This engendered open communication foundational in co-creation processes, encouraging participants to share their ideas and listen actively to others. Involving more colleagues and stakeholders broadens perspectives and enhances the inclusivity of the project.

   "One person or more than one group involved in helping (to) develop a curriculum that makes sense and pulling on the strengths of both sides. Both sides should have input both sides should be involved and pull on the strengths of both sides to get to make it a stronger programme (…)." Ghanaian Faculty

5. Communication and logistics: Common challenges include availability of team members for all meetings, lack of input from some team members, and logistical difficulties in coordinating across different geographic and cultural contexts. These challenges require

effective management and adaptation strategies to maintain project momentum. Clear communication and respecting agreed-upon timelines are crucial in co-creation efforts to ensure smooth collaboration and avoid delays in project progress. Regular online meetings and active involvement of all parties help maintain momentum and timely inputs.

Conversely, faculty also noted challenges in the experience including around the organization of teams, challenges in communication between team members, and the need for more structured support for organization of team meetings. Specific challenges included poor meeting attendance and delayed communication between team members during the summer. Some participants expressed the desire for longer timelines for creation of content. These are likely functions of the 'first time' effect of course design and the tight timelines as noted by some. This theme is summarized as "communication and logistics." Despite these challenges, faculty managed the creation and delivery of the courses due to the enablers listed above.

"We don't know what to do, because what we're expecting to be done has not been done. So, moving forward, I think we should be committed to and respect the time and the standards of each other, where we are doing cooperation." Canadian Faculty

"Usually, they don't want to have long meetings we have here…we realise that their way of thinking since it's quite different from ours, it took some time for them to adapt to our way of thinking and for us to also accept their way of thinking. So maybe the first few were just trying to sort of find your footing, which was why things were delayed a bit. But now things are quite okay." Ghanaian Faculty

Intended Design

Analysis of the co-creation process also gives insight on the intentions of the faculty in their approaches to creating course content and experiences. Some of the issues raised also included informational gaps that may no longer be relevant to future iterations but are noted as potential issues for other short course programs.

1. Appropriate learning emerges from co-creation: Faculty felt that co-creation allowed them to identify relevant content and learning activities that made the courses appropriate for the learners. The process of adapting knowledge to the Ghanaian context through co-creation was felt to be a significant contributor to the success of the short courses. Notably, participants felt that co-creation resulted in a strong emphasis on incorporating practical, hands-on activities in the educational programs. This is an interesting intention given the learner experience data below that desired more practical experiences (see section on Course Experience). Other pedagogical elements tied to the co-creation process include the use of technology and interactive learning activities.

"I think that's reflected in the materials; it definitely is reflected in the actual teaching. But obviously, (…) it's critical for material development in order to make it more contextually relevant for the participants." Ghanaian Faculty

"I'm super thrilled that I joined. It's been fantastic to learn both ways. I think there's definitely things that we can take back and we can change from what we've learned, and then hopefully, hopefully the other way too, so just looking for people to feel good and comfortable and to help all patients." Canadian Faculty

2. Challenges in Content Tailoring: One of the major challenges noted during design is the unique difficulty in tailoring educational content to the heterogenous audience enrolled in some of the courses. This lack of specific information about the learners was felt as a challenge in selecting appropriate content and identifying the level of depth necessary for the learners. This was perceived as a particularly novel challenge for this iteration of the courses that was not faced in previous education experiences.

   "I understood there would be a broader range of people, people that weren't involved in health, so much, from the initial emails and discussion, that there may be taxi drivers, or Uber drivers or truck drivers or other people I didn't - we didn't know. So, trying to develop the course content, knowing that the range of audience can be like, very, very wide, was a challenge, which I think we tried to do." Ghanaian Faculty

3. Adaptability: Given that this was the first iteration of the courses, faculty recognized they would need to be adaptable during teaching to ensure the material is accessible and effective for all participants. This was felt to be mirrored by the experience of co-creation and also a regular part of delivering education in a global health context.

   "I think that it was more of what we have to adapt. (…) one of the things I realised was (that) the Canadians were very much interested in case studies, okay. And we had some cases, but we realised that they really wanted practical cases as part of the training. So, we tried, (…) we also decided that since what two of Ghana team members were on the field, we would rather let them finish [with] day-to-day challenges, instead of us still talking about what we have known maybe 10 years ago or even 15 years ago." Ghanaian faculty

   "And then in terms of the delivery, I think, I think we just need to allocate a bit more time for certain topics. And then some others need to be a little shorter. But it's hard to say that

because the students have loved. And it's been a lot of discussion that kind of makes things longer." Canadian Faculty

Learner experience

The intended design from the co-creation process led to a successful learner experience, in that course content anticipated knowledge gaps of learner and allowed for adaptable teaching. This is evidenced by the learners almost uniformly reporting a positive experience. They reported high satisfaction with teachers and gratitude for the opportunity. While a social desirability bias cannot be ruled out in evaluation surveys, the consistent pattern of positive results and triangulation with focus group data indicates the high level of satisfaction to likely be a stable finding. Evidence of this also includes a common sentiment across the courses beyond the evaluation's scope: expand training and access to other regions and colleagues. Consistently high reporting of satisfaction across survey items also limits the value of inferential statistics due to low variance.

Overall Summary of Learner Experience

High rates of satisfaction and recommendation to peers: All courses received high marks for satisfaction and the likelihood of participants recommending them to peers (See Table 2).

Requests for more practical applications: A common theme across all courses was the request for more practical, hands-on learning experiences.

Engagement and safety in learning environment: Participants consistently noted the courses were engaging and provided a safe and supportive learning environment.

Effective pedagogical approaches: The courses were praised for their effective teaching methods and the ability to meet learning objectives.

Table 2: Learner Experience Summary by Course

	Community Emergency Care (CEC)	Emergency Preparedness and Response to Epidemic Prone-Diseases (EMPRESS)	Prehospital Emergency Care (PEC)	Palliative Care	QI
# Responses	21	19	19	20	22
Satisfaction	High levels of satisfaction and likelihood of recommending the course to peers were reported.<br><br>90% were Extremely Satisfied with the course overall and 95% would be Extremely Likely recommend to a colleague	High satisfaction and effective pedagogical approaches were noted.<br><br>90% were Extremely Satisfied with the course overall and 95% would be Extremely Likely recommend to a colleague.	High satisfaction; effective in meeting learning goals.<br><br>89% were Extremely Satisfied with the course overall; 100% would be Extremely Likely recommend to a colleague.	High satisfaction, with the course described as highly effective.<br><br>85% were Extremely Satisfied with the course overall; 100% would be Extremely Likely recommend to a colleague.	High levels of satisfaction, with many participants wishing to return for future learning.<br><br>86% were Extremely Satisfied with the course overall; 100% would be Extremely Likely recommend to a colleague.

Engagement	Participants found the course engaging and felt safe in the learning environment.<br><br>95% Strongly Agreed that the classroom was effective, welcoming and inclusive.	The course was described as engaging and inclusive.<br><br>100% Strongly Agreed the classroom was effective, welcoming and inclusive.	Positive environment with supportive learning atmosphere;<br><br>100% Strongly Agreed the classroom was effective, welcoming and inclusive.	The course included a variety of teaching methods including lectures, group discussions, and role-playing.<br><br>95% Strongly Agreed the classroom was effective, welcoming and inclusive.	The course was considered engaging and supportive of a positive learning environment.<br><br>82% Strongly Agreed the classroom was effective, welcoming and inclusive.
Suggested Improvements	Increased practical applications, more course time, and access to slides.	More practical applications, extended training time, and post-training slide distribution.	More hands-on activities and wider participation across Ghana.	Follow-up courses and further integration of palliative care into regular healthcare training.	More interactivity, practical application of knowledge, and better access to slides.
Key learnings from participant perspectives	Emphasis on algorithms for care such as ABCDE.	Importance of field exercises and practical application of course content.	Specific procedures and emergency care techniques.	Principles of palliative care, pain management, and ethical issues.	Importance of QI in healthcare, application of QI tools, and leadership in QI efforts.

Participant quotes	*I would like to thank the teachers for effortlessly teaching and equipping us with great knowledge. Much appreciated*	*I am super grateful for the opportunity and knowledge gained and follow up on us as though we had a viral Hemorrhagic fever...so we can impact our communities with the knowledge you gave us. Thank you*	*The delivery of the module and practical sessions were apt and very interactive*	*I would like to thank them very much for the time and commitment. God bless you all beyond your imagination. I now know how to care better for my aged family members and clients as well as others who would need palliative care.*	*The delivery was very interactive and impactful, and this method of learning should be inculcated in other courses*

Focus Group Themes: Evidence of Pedagogical Effectiveness and Learning Experiences

Focus groups held with learners across both the Palliative Care and QI courses reflected important learnings about the experience and initial outcomes from the first modules of the courses. Across both groups, learners felt the impact of education on their professional/clinical practices and appreciated the longitudinal approach across both modules. For example, one palliative care participant noted: *"so I think the interactive sections [were] very helpful…and it builds upon how we are supposed to go about it."* Emergent issues around the ability to enact/transfer learning in their home contexts were noted by some learners. These included greater engagement with local stakeholders and alignment with the institutional resources, expertise, and authority to enact their knowledge from the courses. Table 3 and 4 highlight the themes and exemplars from Palliative and QI respectively.

Table 3: Palliative Care Themes

Theme	Description	Quote
Enhanced Patient and Family Engagement	Learners reported feeling better equipped with the necessary skills to involve patients and their families more deeply in the care process. This is evident from improved discussions about care expectations and outcomes, which foster a supportive environment for both patients and families.	"Points we did advance care plan for the person by him helping him make his final decision by changing his will, he was able to reconcile back to the family… then family or relatives were also brought in to understand the condition of this man" Participant FG1
Strengthened Communication Skills	Learners reported improved ability to communicate effectively across various stages of palliative care. This included better articulation of medical conditions and care plans, which helps in reducing misunderstandings and enhancing the comfort of patients and families.	"So, I came to understand communication really helps - when you tell patients what they're going through…So I started educating patients even though I was alone" FG2
Targeted Education and Ongoing Learning	The module broadened learners' understanding of palliative care.<br><br>It also provided confidence and encouragement to apply skills into practice or take advantage of existing tools.	"In my facility, we use the electronic medical record, even though we had pain [scales] in it, we're not really using it. But after the course I tried it out using the pain scale at the OPD even before they go to the ward." FG2

Advanced Pain Management Techniques	Participants felt the education introduced them to new approaches to pain management strategies, incorporating both pharmacological and non-pharmacological methods. Participants reported applying these new skills in creating specialized teams and protocols to manage and alleviate pain more effectively, thereby improving patient quality of life.	“You know, on our wards normally when patients call out in pain or requests for pain medication, most healthcare providers have the mindset that a patient might be getting addicted…Pain is what the patient says it is. And then you should try as much as possible to alleviate the pain of that patient.” Participant FG1
Ethical Practice and Confidentiality	The training has reinforced the importance of ethical considerations and client confidentiality in palliative care. Participants felt improved competency in handling sensitive information and making ethical decisions that respect the dignity and wishes of patients and their families.	“But after the palliative course, what I took home from it, they helped with my practices, even when you're talking with the religious [advisors] are involved in them, you make sure that certain information is not shared” FG2
Overcoming Palliative Care Challenges	The educational sessions prepared participants to tackle common challenges in palliative care, such as resistance from other healthcare staff and the integration of complex care strategies. Participants also felt it enhanced their ability to manage end-of-life care sensitively and effectively, focusing on family reconciliation, advance care planning, and legal considerations.	“So I think, prior to taking this course, it had not occurred to me, like myself, and then the team I work with, to sometimes even involve lawyers have our patients who have the legal team, to incorporate them into a system, we're just focused on giving the health care and all that…So we actually pulled his lawyers to his bedside. And then he didn't have the strength to sign and document things. But then he could speak. So, the lawyers recorded him they had, they had a video of him” FG1

Organizational Alignment and Institutional Change	Participants felt one of the major challenges in enacting their knowledge was the need for greater upskilling of colleagues, recognition of their expertise and roles, and greater resources for palliative care. Engagement of facilities in which they worked or had connection to, would be important to enhance the impact they can have as participants. Some suggested engaging local stakeholders in identifying future course participants and making more formal relationships with local institutions and health organizations.	“After we are done with your course the facilitators, or the organizers must get in touch with the various facility heads…you're not a key stakeholder in the facility. So, whatever you've learned, you can't really implement it.” FG1

Table 4: Quality Improvement Themes

Theme	Description	Quote
Participant Impact and Knowledge Gain	Participants of the QI course reported a significant gain in knowledge and an increased sense of empowerment. They reported feeling more confident in addressing QI issues within their facilities.	"And I was amazed with this course that quality isn't only about the bigger changes, but the little things. So, I felt that I have a huge responsibility to look at even the little things in the facilities and try to make the little things to make the bigger changes. So, you don't have to wait for you to make a bigger impact, you need to start from the little." FG1<br><br>"Okay, so, at the end of module one, I felt emboldened that I don't have to be part of management to effect a change in my facility, I can start in my small corner, which can resonate with my department, and then the hospital as a whole." FG2
Enhancing Stakeholder Engagement	The course emphasized the importance of stakeholder engagement and communication. Learners felt they could engage directly with stakeholders to understand their needs better and appreciate their efforts.	"Communication is part of client satisfaction, if you want our client to appreciate what we are doing, we have to communicate with them." FG2
Leadership and Team Empowerment	The course fostered leadership skills and encouraged participants to lead by example, which inspired colleagues to be motivated and time conscious. The implementation of QI plans involved not just individual effort but also a collaborative approach, engaging various stakeholders and tailoring communication to resonate effectively with management and staff.	"During our leadership session, we realised that we learned some traits or characteristics of who a potential leader should be. And like I said, being in the mud with people, so you try to drive your people along with the change." FG2

Practical Application and Continuous Connection	The hands-on approach in the course was felt to help translate theoretical knowledge into practical skills. Participants discussed ways to remain connected post-training, such as using digital platforms for sharing success stories and challenges, which aids in continuous improvement and implementation of new ideas. They felt they would appreciate continued opportunities afforded by the short course program for these types of connections.	"WhatsApp group is now a very effective to remember when we did a training, we put the information on the page at all for other colleagues" FG1<br><br>"But another step that we could introduce could be, for example, if my hospital QI team is going to meet, or we have an activity, we can decide to extend an invitation to assignment to come and join our meeting, or make a presentation, or zoom. Yes, it's possible. Yeah, to reduce costs, or zoom. And then there's that kind of blend, then we tell you what we want you to talk about, just so that you can inspire our team" FG2<br><br>"I've been doing QI training before and until I came here, I didn't know how to do a proper PDSA, seriously. But I think I now have a fair idea. I think the course content covers a whole lot other than just focus on this. Core key items. So they mentioned a bit on leadership and seriously, the person who presented on the leadership and the way he went about presenting on the leadership…I was telling my people I could sit down and listen to him all day and never get bored because I made it so practical, and he made us all understand that we are all leaders." FG1

Addressing Challenges in QI Projects	Participants reported better in anticipating and in addressing potential challenges such as resistance to change, increased workload, and resource constraints. Effective communication and stakeholder engagement were emphasized as crucial lessons for overcoming these barriers and successfully implementing QI projects.	"We realised that to create the quality improvement culture, people need to understand quality.<br>And then they [stakeholders] need to share your vision. When you allow people to understand the reason why you're doing certain things they buy into it. So, I think I learned that in the about letting people understand your vision" FG2
Infrastructure and Resource Allocation	Issues like infrastructure needs and transportation for reaching out to the community were highlighted. The importance of teamwork and support from community leaders was underscored for the successful implementation of new processes for QI and the ultimate success of any project	"The challenge is because we are working with people and people are dynamic, people are unpredictable. You can't predict that, okay, I know, this is what I'll do. But tomorrow, you can imagine that people are not like that again." FG2<br><br>"One major support will be the community. We'll have to take that up seriously… we've come here we've learned that when you identify a problem we don't just stand up and then try to solve the problem needs to sit down analyze, make an HTS means the PPM PDSA, all those things. So together as the team needs to work as a team, it's not a one man's job." FG1

Summary of Learner Experience

Intended Processes & Outcomes

Overall, from the learner perspective, the pedagogical design and delivery of the short courses met the intended goals of each course and the overall program. Across all courses, participants reported feeling highly satisfied, grateful, and knowledgeable in the goals of the courses as expected.

Emergent Processes & Outcomes

The emergent process and outcome learnings from this evaluation identify opportunities to make the emergent or unintended, explicit, and intentional.

1. Connections & Relationships: One of the notable emergent processes from this data is clear evidence of 'cohort effects.' Most pronounced in the QI course, learners reported good connections and emerging relationships with one another that they wished to sustain. Use of technology such as WhatsApp and other media is expected to facilitate these connections. One of the beneficial outcomes is the close connection made by individuals who would otherwise not have an opportunity to meet or network.

2. Longitudinality: The modular nature of two courses allowed for longitudinal development of ideas and transition from theory to practice as course participants complete their practical project. One of the 2023 QI cohorts was invited to present their QI project at a national Christian Hospital Association of Ghana (CHAG) conference on reducing stillborn rates in 2024.

   While this is also an intended process, the consequent emergent learnings for participants who are working to put into place specific learnings from Module 1, made them better prepared to benefit from Module 2. A partial explanation for the success of longitudinality of course is greater time and contact with faculty. Learners indicated that two modules allowed for a transition from theory to practice and a period of reflection, consolidation, and potential application of knowledge between modules. This helped mitigate the major emergent issue discussed below.

3. Theory to Practice: A major process issue for teaching as identified by participants was greater hands-on and practical experience. While certainly many courses did provide practical learning opportunities and exercises (and noted by several participants), this remains a common theme across all courses. Palliative and QI courses benefited from longitudinality which allowed more time for practical issues to be discussed and an opportunity to transition from theory to practice. In the other courses, participants had different ideas on how to manage the practical/applied context of learning including field

visits, simulations, and greater engagement of colleagues or peers. From a pedagogical perspective, it is expected that new learning or knowledge will lead to learners identifying new conceptual and procedural gaps in the curriculum. As such, the learners expressing a desire for more practical experiences at the end of training is not a novel or unusual finding and is also an indicator of course success.

Faculty facilitator experience

Faculty facilitators across all courses confirmed many of the perspectives of learner participants. Facilitators recognized many of the same benefits and issues identified by learners including the likely implementation challenges and the need for more practical experience. In doing so, they acknowledged some of the limitations imposed on the short courses by resources and time.

1. Adapting to Diversity & Tailoring Education: Faculty from both Canada and Ghana acknowledged challenges in understanding and adapting to the diverse backgrounds and skill levels of healthcare professionals during the teaching itself. There is an emphasis on tailoring course content to meet the specific needs and contexts of different groups, aiming to make training relevant and applicable. In international settings, understanding and integrating local cultural values, local diversity and norms are crucial. This adaptation ensures that the training is not only effective but also respectful and appropriate to the local context.

   "(…) something that I had to adjust to, after my first day, was understanding the different contexts that we both come from, the Canadian team versus those who work in Ghana, and adapt some of our examples to reflect more local issues, rather than things that we may deal with in Canada… I think what stood out to me was the significant variations in the facilities (settings) that the participants came from. (…) [They included] four team members from one facility, some from Northern Ghana, some from more central Ghana, different regions (…). And just based on that, (…), it really speaks to the kind of variation in progress for quality improvement in different kinds of facilities." Canadian Faculty

2. Practical and Hands-On Training: Facilitators reflected on the need for practical skills through hands-on training and noted the use of simulation equipment and real-life scenarios in some courses. Trainers and course developers discussed the potential difficulties participants might face in implementing learned skills in real-world settings. Faculty noted that participants will need ongoing support and mentorship to help overcome these challenges which the course structure was not set up to provide.

“I just think if we had more simulation equipment and simulation space or readiness, (…) I think that might optimise the course even further. It's good as it is (…). But if we were able to enhance the simulation component, I think that would go a long way.” Canadian Faculty

“So, we need to do more to make sure the knowledge reaches a lot more people. (…) a [learner] was asking me, she wants to train people at the [local] facility. (…) she would like me to come down to a facility so that more of its colleagues can [learn] (…) because she has seen that what we're teaching is very impactful” Ghanaian faculty

3. Logistics and Future Planning: Logistical challenges such as scheduling, technology, room setup, and resource were mentioned but did not interfere with teaching experience. Suggestions for future improvements include more time for planning and anticipating technical or logistical issues. Time management and scheduling were particularly mentioned. However, faculty also felt very well supported by KNUST and the organization team.

“Well, I think that for example, yesterday I'm struggling with a network. And this is basically the location. So, then we'll talk, we'll tell them, and they do bring additional things such as with the projector quality. And so, either we'll make it more pronounced, or we get sharper projector. I mean, these are small, small fixable things, not nothing major is the kudos to that organisation” Canadian faculty

“(…) it's really a blessing. (…) the opportunity to also be part of it and contribute to it. (…) the team of KNUST and Toronto - University of Toronto, people are awesome. The working relationship was great. And I think that they should hear so that they keep that up.” Ghanaian Faculty

“We know that the equipment that we use is a bit limited (…) but even that they resource pretty quickly. And (...) we also brought some things. So, I think in the future, maybe we just had to work (…) on that part a little bit more, but anything that we requested (…) they brought in, (..) overall, like very, very well run.” Canadian faculty

4. Purpose and Engagement: Instructors felt a sense of purpose in their work, recognizing the value of their contributions to healthcare quality by equipping professionals with the necessary skills and knowledge. They also reported a high sense of engagement from participants in how they responded to both Canadian and Ghanaian faculty including the questions and examples offered during course time.

"Everybody is participating, everybody's willing to share the experience even when it is even a failure. They are ready to share, and it inspires me a lot because it now tells me that (…) people really are looking for change and how they can make changes in the system"
Ghanaian faculty

Integration of Results

The evaluation data speak to multiple perspectives and emergent themes. One possible way among many of integrating these results is through the 2x2 matrix offered by Haji et al.[2] as seen in Table 5. This framing allows us to see that the short course experiences were successful in meeting intended processes and outcomes and highlight emergent issues faced by learners and faculty. Notably, the key issues noted by course designers and faculty focused on organization, a need for extended timelines, and the need for more information to determine what to teach and the depth of that teaching. However, given the first run of courses has been completed, it is likely that these issues may be already addressed. Some technological issues were noted during course delivery though these were ably addressed by KNUST staff; all faculty felt very supported during the experience.

From the learners' perspective, the short courses were experienced as very effective learning opportunities which presented new knowledge but also instilled a desire for greater experience and practical training. Moreover, learners recognized that the ability to influence change and impact the healthcare system would depend on the coordination of colleagues, leaders, and other stakeholders in the healthcare system. As such, learners felt the knowledge from these courses would need to be disseminated broadly for the impact on the larger system practice to be felt.

Table 5: Framing of Results into Emergent and Unintended Processes & Outcomes

	Planned	Emergent/ Unintended
**Process**	Interactive, practical, and contextually relevant education for Ghanaian primary care<br><br>Distillation of new knowledge for diverse audiences	Connections and relationships built between participants, facilitators, and designers which can be leveraged for future education<br><br>Desire for further practical and applied learning opportunities<br><br>Organization and timeline of co-creation<br><br>Logistical/support issues during course delivery
**Outcome**	Delivery of inaugural short courses: pilot phase<br><br>Relevant knowledge with high participant satisfaction and perception	Recognition of system changes/effects required for successful implementation of short course knowledge<br><br>Recognition of ongoing and practical learning needs

Lessons Learned

From the comprehensive evaluation of the collaborative educational program led by Kwame Nkrumah University of Science and Technology and the Department of Family and Community Medicine, several key lessons have been learned that are pivotal for future educational courses in the Africa Higher Education Health Collaborative:

1. Importance of co-creation: The co-creation process, though challenging, proved essential in harnessing diverse insights and expertise, ultimately enriching the educational content. The engagement of multiple stakeholders led to a more tailored and contextually relevant learning experience. Moving forward, structuring co-creation processes with clear

communication and defined roles will enhance the effectiveness and efficiency of program development.

2. Flexibility and Adaptability in Course Design and Delivery: Flexibility in adapting course content and structure to the varying backgrounds and skills of participants was crucial. This adaptability needs to be maintained and enhanced in future programs to accommodate the diverse needs of participants and to handle unforeseen challenges more effectively.
3. Effective Engagement and Communication: Regular and structured communication among course designers helped in maintaining project momentum and alignment. Future programs may require more time for co-creation, engagement in team meetings, and clarity on roles and responsibilities.
4. Building on Success and Addressing Practical Needs: The high levels of participant satisfaction and engagement indicate that the courses met their primary educational goals. A recurring theme across the courses was the need for more practical, hands-on learning experiences. This feedback underscores the importance of integrating more simulation-based/field work and interactive components to continue to bridge the gap between theoretical knowledge and practical application.
5. Longitudinal Impact and Stakeholder Coordination: The longitudinal nature of some modules allowed for the development of ideas and better preparation for practical application. Future programs should consider the benefits of extending this approach to foster deeper learning and more sustained impact.

These lessons form a foundation for refining future educational programs and suggest a model of continuous engagement and improvement that can be adapted to other contexts, enhancing the overall quality and impact of other co-creation-based education initiatives.

References

1. Acheampong, Kulasegaram, Mensah, Ndiaye, Owusu-Ansah, Owusu-dabo, Owusu, Rodas, Rouleau, Wilson, Wilson. The Concept of Co-Creation: Strengthening Primary Health Care (PHC) in Ghana through an Innovative North-South Partnership. Manuscript submitted for publication. 2024.
2. Haji F, Morin MP, Parker K. Rethinking programme evaluation in health professions education: Beyond 'did it work?'. Med Educ. 2013 Apr;47(4):342-51.
3. Kahlke RM. Generic Qualitative Approaches: Pitfalls and Benefits of Methodological Mixology. International Journal of Qualitative Methods. 2014.

Appendices

A. Study Protocol
B. Methods Summary
C. Ethics Approval KNUST
D. Evaluation Questionnaire and Tools

Appendix A : Study Protocol

EVALUATION TARGET	Questions:	Data Sources	Collection Approach	Considerations for Course Adaptation
Co-Creation Approach	1) What is unique and novel about the co-creation approach used to deliver the course?<br>2) What are the advantages of this approach and what are the challenges?<br>3) What do other teams need to know?	Course leads and program leads; education materials, minutes, course outputs	Interview (priority)<br>Review of materials (secondary)	Interview course leads specifically about their course
Course Experience	1) What is the experience of learners in each course?<br>a. Satisfied with teacher<br>b. Satisfied with the course content<br>c. Satisfied with how it was delivered<br>d. How relevant was the content to the work of the learner?<br>e. Any key moments, activities, or things that were perceived to be meaningful or effective?<br>f. Improvements/Strengths<br>2) What is the experience of teachers in each course?<br>a. What seemed to reach students well? What didn't work well?<br>b. How did you adapt course to emerging learning needs (if at all)?<br>c. Did they feel supported by the program and staff?<br>d. What were points of friction in their classroom experience?	Learners, teachers, and staff; Observation of classroom activities	Questionnaire of Learners (priority- a-c, e)<br>Interview or focus group of learners (d & e)<br>Focus group of teachers<br>Interviews with Staff	Global questionnaire for all courses with 3 to 4 specific questions for each course<br>Focus group by course; common and unique questions

	3) What insights do staff who promote, support, and coordinate each course have about course experience?			
Learning Outcomes	1) What is the knowledge gained by participants at the end of the course?<br>2) How is the knowledge mobilized to activities outside of the current course (next course, their clinical work, back in their community)?<br>3) How does this knowledge align with their social and community context?	Learners; formal assessments and knowledge artifacts created in the classroom	Survey of participants (at the end of the course and a few weeks later)<br>Interviews	Similar approach for all courses; take advantage of multiple courses in the same domain to track learning (i.e. Palliative Care)
System Level Impact and Change	1) Overall, what are the lessons learned from this experience for the Collaborative?<br>2) How will KNUST sustain this program going forward?	Program Leaders	Emergent/Interviews	N/A

Appendix B: Methods Summary

Target	Type of tool	Target respondents	Number of respondents	Who will collect data	Analysis	Timelines
Co-Design	Interviews w/ course leads	Course designers	8-10 (2 per course)	RA	RA w/input from OES	Longitudinal through courses
Learning Experience	Questionnaires	Learners	Dependent on enrollment; aim for 60%+ response rate	Online Survey (DFCM)	OES	
Learning Experience	Interviews	Teachers	Depends on # of teachers, aim for at least 2 teachers (1 DFCM, 1 KNUST) per course	RA (KNUST)	RA w/input from OES	After each course
Learning Experience	Observations	Observers (Marie, Ernest, Jennifer)	1-2 observations during the first course	Project team	Observers & RA	During Course
Learning Outcomes	Questionnaires+	Learners	Dependent on enrollment; aim for 60%+ response rate	Online Survey (DFCM)	OES	After each course
Learning Outcomes	Focus group/Group interviews*	Learners	2 focus groups per course (6-8 for focus groups; 2-4 for group interviews)	RA (KNUST)	RA w/input from OES	Prior to 2nd course for Pal/QI

Appendix C: Ethics Approval

Kwame Nkrumah
University of Science
and Technology, Kumasi

College of Health Sciences
SCHOOL OF MEDICINE AND DENTISTRY

COMMITTEE ON HUMAN RESEARCH, PUBLICATION AND ETHICS

Our Ref: CHRPE/AP/126/23

23rd February 2023.

Dr. Princess Ruhama Acheampong
Department of Health Promotion
Education and Disability Studies
School of Public Health
KNUST-KUMASI.

Dear Madam,

LETTER OF APPROVAL

Protocol Title: ***"The African Higher Education Health Collaborative: A Baseline Needs Assessment Research Study."***

Proposed Site: ***School of Public Health, KNUST.***

Sponsor: ***Mastercard Foundation.***

Your submission to the Committee on Human Research, Publications, and Ethics on the above-named protocol refer.

The Committee reviewed the following documents:

- A notification letter of 24th January 2023 from the School of Public Health (study site) indicating approval for the conduct of the study.
- A Completed CHRPE Application Form.
- CHRPE Participant Information Leaflet and Consent Form.
- Research Protocol - Consolidated.
- HEMP and HECO Interview Guide for Leaders, Learners and Employers.
- HENT Interview Guide for Leaders, Learners and Ecosystem.
- HEMP and HECO eSurvey for Employers, Leaders and Learners.
- HENT eSurvey for Leaders, Learners and Ecosystem.

The Committee has considered the ethical merit of your submission and approved the protocol. The approval is for a fixed period of one year, beginning **23rd February 2023** to **22nd February 2024** renewable thereafter. The Committee may, however, suspend or withdraw ethical approval at any time if your study is found to contravene the approved protocol.

Data gathered for the study should be used for the approved purposes only. Permission should be sought from the Committee if any amendment to the protocol or use, other than submitted, is made of your research data.

The Committee should be notified of the actual start date of the project and would expect a report on your study, annually or at the close of the project, whichever one comes first. It should also be informed of any publication arising from the study.

Thank you for your application.

Yours faithfully,

Rev. Prof. John Appiah-Poku.
Honorary Secretary

Appendix D. Evaluation Questionnaire and Tools

Tool #1: Semi-structured Interview Course Designers

Evaluation Target: Co-Creation Approach

Population: Course Designers (DFCM & KNUST)

Description: A semi-structured interview with DFCM and KNUST Faculty.

Semi-structured Interview Guide:

1. Tell me about your role in the KNUST-DFCM Collaborative.
2. What was your experience of creating the course like? What did you like about it? What did you struggle with?
3. What does co-creation mean to you? Probe: How do you understand the concept? What did you have to do differently from your previous experiences? What is unique about it?
4. What skills or ways of thinking did you have to adapt in order to engage in the course creation process?
5. How do you think co-creation will impact the learning experience in Ghana? What things were 'created' that do you anticipate will impact learning in a positive way (in your opinion)?
6. What is the importance of co-creation for your work?
7. What do others who may be unfamiliar with co-creation have to learn in order to successfully engage in it?
8. Anything else you would like to tell us?

Tool #2 : Learner Questionnaire

Evaluation Target: Learning Experience

Population: Learners

Description: Questionnaire

Questionnaire for Participants

Demographics

1. What is your gender?
   Male___ Female___ Prefer not to say___

2. How old are you?

3. What district/municipality/metropolitan area are you from?

4. What cadre or professional role to you belong to?

➢ Physician, Nurse, Midwife, Healthcare Assistant, Health Officer, Other

5. How long have you been in your current role?

   <5 years 6-15 years >16 years

6. How long have you worked in healthcare?

   <5 years 6-15 years >16 years

Course Entry & Recruitment

7. How did you find out about this course?

➢ Email advertising, website, social media advertising, recommendation form a colleague or supervisor,

8. How easy was it to find information about this course?

   1-Very Easy 2-Somewhat Easy 3-Somewhat Difficult 4 -Very Difficult

9. How easy was it to register for this course?

1-Very Easy 2-Somewhat Easy 3-Somewhat Difficult 4 -Very Difficult

Course Experience & Outcomes

10. Overall, how satisfied were you with the learning experience in this course?

1-Extremely Dissatisfied 2- Somewhat Dissatisfied 3-Neither satisfied nor dissatisfied 4- Somewhat Satisfied 5-Extremely Satisfied

11. Overall, how well did the teachers teach this course?

1-Not well 2- Slightly Well 3-Modaretly Well 4-Very well

12. How likely are you to recommend this course to a colleague?

1-Extremely unlikely 2-Somewhat unlikely 3-Neither Likely nor Unlikely 4-Somewhat Likely 5-Extremely Likely

13. Please indicate your agreement with the following statements

1-Strongly Agree 2 Agree 3-Neither agree or Disagree4-Disagree 5-Strongly Disagree

- I could understand course content easily
- The resources were helpful in my learning
- I could understand the teachers easily
- I felt comfortable asking questions in class
- I felt the classroom was welcoming and positive
- The small group activities were helpful for my learning
- I enjoyed learning with my colleagues
- The course content was relevant to my professional work
- The course objectives were clearly stated
- The course met the stated objectives
- The course met my professional learning goals
- I feel confident that I can apply the course content to my own work
- I have a plan to implement the course content in my own work
- I can identify the challenges to applying the course content in my own work
- My supervisor is supportive of my using course knowledge in my own work
- I know more about the course topic than when I started
- I plan to take future courses at KNUST that are not related to the current course

For Module 2 that builds on previous modules

- ➢ The course built on the previous module
- ➢ The previous module prepared me to learn in the current module

14. Please list up to three things that you felt you learned as a result of this course. Please use point form or short sentences if you prefer.

15. Are there any questions or doubts you would like clarified about the course content? Please use point form or short sentences if you prefer

16. Please list up to three things you would like to change or improved in this course. Please use point form or short sentences if you prefer.

17. Is there any feedback or comments you may want to share with the teachers and course leadership?

Tool # 3: Teacher Interview

Evaluation Target: Learning Experience

Population: Teachers

Description: Focus Group/Interview Guide for Teachers/Instructors

1. What was the experience of teaching like for you? *Follow-up: did you enjoy it? What were your surprised by in the learners and the teaching experience?*

2. Are there any moments or interactions with students that stand out for you? *Probe: Any moments you felt that you and the course were being successful, any moments you felt were challenging or difficult?*

3. What do you think the students learned? *Follow-up:* What do you think in the course and/or your activities that helped them learn that?

4. What do you expect the students do with this learning? Where do you think they might struggle or be challenged by?

5. How did you find the support and logistics for this course – for example the classroom setup, any tech issues, scheduling and administrative support from staff?

6. How did you adapt or modify the course from what you expected to do?

7. Was there anything you wish you could do differently either in the planning or delivery of the course? *IF Yes* – What is it? And how do you plan to address it in future courses?

8. Is there anything else you wish to tell us about your experience?

<u>Tool #4: Learner Interview</u>

Evaluation Target: Course Outcomes

Population: Learners returning for Module 2 of QI or Palliative Care Course

Description: Focus Groups for Returning Students (Module 2 for QI and Palliative)

1. Can we confirm you took X course at KNUST on XXX date?

2. How did you feel at the end of course? What were your main takeaways?

3. How does what you learned in the course influence your typical day? *Probe:* Maybe you can begin by describing your responsibilities and typical workday.

4. Tell me about a recent example in your work in which you used what you learned in the course. Be specific as possible. *Follow-up:* Was there something specific in the course or teaching (example, an activity, lecture, reading, or interaction with a teacher) what was important in helping you use the course knowledge. *Follow-up:* How did you feel about this example? What did you adapt or change from what you learned in the course?

IF Answer is NO: Are there any examples or experiences you can think where you *could* have applied course learning? *IF Answer is NO:* Tell me more about why not. For example, is it because there were no instances where the course learning was relevant or were there any challenges (e.g. time, technology, support) that prevented you from using this knowledge?

4a. *If not raised at all,* what were things that helped you apply your knowledge? What were challenges?

5. Were there any surprising or unanticipated ways in which the course influenced your work or those of your colleagues or your leaders? *Possible probes: Use an example. E.g. did the knowledge you learned about palliative care apply to other clinical areas? E.g. Maybe you met some peers or colleagues who have become part of your professional network or support system? Etc.*

6. Now that you have had a chance to reflect since the end of the course, overall, what would you say was most effective for you?

7. What would you want changed or modified in the course? *Possible: the registration, organization, content, the teaching style, breaks, timing & logistics etc.*

8. Is there anything else you would like to share?
